# Aberrant Awake Spontaneous Brain Activity in Obstructive Sleep Apnea: A Review Focused on Resting-State EEG and Resting-State fMRI

**DOI:** 10.3389/fneur.2020.00768

**Published:** 2020-08-11

**Authors:** Yue Wu, Wenrui Zhao, Xinyuan Chen, Xiaoyong Wan, Xu Lei

**Affiliations:** ^1^Sleep and NeuroImaging Center, Faculty of Psychology, Southwest University, Chongqing, China; ^2^Key Laboratory of Cognition and Personality of Ministry of Education, Chongqing, China; ^3^Key Laboratory for NeuroInformation of Ministry of Education, Center for Information in Medicine, University of Electronic Science and Technology of China, Chengdu, China

**Keywords:** obstructive sleep apnea, resting-state, electroencephalography, functional magnetic resonance imaging, brain activity

## Abstract

As one of the most common sleep-related respiratory disorders, obstructive sleep apnea (OSA) is characterized by excessive snoring, repetitive apnea, arousal, sleep fragmentation, and intermittent nocturnal hypoxemia. Focused on the resting-state brain imaging techniques, we reviewed the OSA-related resting-state electroencephalogram and resting-state functional magnetic resonance imaging (rsfMRI) studies. Compared with the healthy control group, patients with OSA presented increased frontal and central δ/θ powers during resting-state wakefulness, and their slow-wave activity showed a positive correlation with apnea–hypopnea index. For rsfMRI, the prefrontal cortex and insula may be the vital regions for OSA and are strongly related to the severity of the disease. Meanwhile, some large-scale brain networks, such as the default-mode network, salience network, and central executive network, play pivotal roles in the pathology of OSA. We then discussed the contribution of resting-state brain imaging as an evaluation approach for disease interventions. Finally, we briefly introduced the effects of OSA-related physiological and mental diseases and discussed some future research directions from the perspective of resting-state brain imaging.

## Introduction

As the most common sleep-related breathing disorder, obstructive sleep apnea (OSA) is characterized by excessive snoring, repetitive episodes of apnea, and arousal during various sleep stages. It can lead to severe sleep fragmentation and intermittent nocturnal hypoxemia (1, 2), which may result in excessive daytime sleepiness and increase the incidence of diabetes, hypertension, congestive heart failure, stroke, and cardiovascular disease (3–5). Existing epidemiological studies indicate that OSA is highly prevalent; ~1 billion people in the 30- to 69-years age group may be affected by OSA, and the prevalence rate exceeds 50% (6, 7). Considering the striking prevalence of this disorder in the general population, the underlying neuro mechanism, however, remains largely unknown.

Over the last two decades, different kinds of techniques, including functional magnetic resonance imaging (fMRI), electroencephalography (EEG), positron emission tomography, magnetoencephalography, and functional near-infrared spectroscopy, have been widely used to investigate the neurophysiological characteristics of OSA. And the EEG-derived polysomnography (PSG) has been considered as the gold standard in the clinical diagnosis of sleep disorders. Among these techniques, EEG and fMRI can collect and analyze data more efficiently, which may have greater application value in the clinical diagnosis of diseases.

With considerable development and wide application of neuroimaging technology, more researchers use resting-state brain imaging to explore the dysfunction of the patient's brain (8). Resting-state brain imaging, especially resting-state EEG (rsEEG) and resting-state fMRI (rsfMRI), was widely concerned for its convenience in operation and straightforward interpretation. During brain scanning, patients just need to lie down or sit quietly for about 5–10 min. The instructions invite the participants to stay relaxed in a state of mind wandering with eyes closed or keep on looking at a cross with eyes open (9). After the recording, the corresponding brain activity and functional connectivity (FC) can be obtained, which may help the clinicians to diagnose and treat the disease. There are some advantages for resting-state brain imaging. First, resting state requires no task-related stimulation, and it has limited requirements on patient's cooperation and interviewer's experience. It also reduced the influence of some irrelevant variables, for example, the familiarity for experiment materials. Second, the results are not dependent on the experimental paradigm, which is conducive to compare among multigroups or cross-center data (8). Third, compared with whole-night PSG, it requires a relatively short time to record. Therefore, the research on resting-state brain imaging is becoming popular, and many big data platforms have been built from the fields of psychology, neuroscience, and clinical radiology (10).

The aim of our review is to summarize the research progress of OSA in the field of resting brain imaging and make suggestions for further research. First, we summarize different kinds of rsEEG and fMRI analytic methods used in the investigation of OSA. Second, we outline the main modality-specific results of OSA research in EEG and fMRI, respectively. Last but not least, current status and future directions of the research of OSA were prospected from the aspects of comorbidities of OSA and some new emerging techniques, such as EEG-fMRI, machine learning, and comorbidity.

## Resting-State EEG and Resting-State fMRI

Neuroimaging techniques have made tremendous progress in the last two decades. Electroencephalography and fMRI are both non-invasive techniques, and more importantly, both have been installed in many research centers and hospitals. At present, rsEEG and rsfMRI are the most widely utilized techniques. Here we focus our review on these two modalities.

### Resting-State EEG

As a technique to record the electrophysiological activity of the brain, EEG possesses multiple advantages over other techniques, including high temporal resolution, non-invasiveness, and relatively lower costs. Many investigations employed rsEEG to explore its clinical values for diagnosing or treating OSA. Interestingly, both eyes-closed and eye-open conditions are usually recorded for rsEEG. However, the eyes-closed condition is more widely used, because data in this condition are less contaminated by eye-blinking artifact (11).

**Figure 1:**
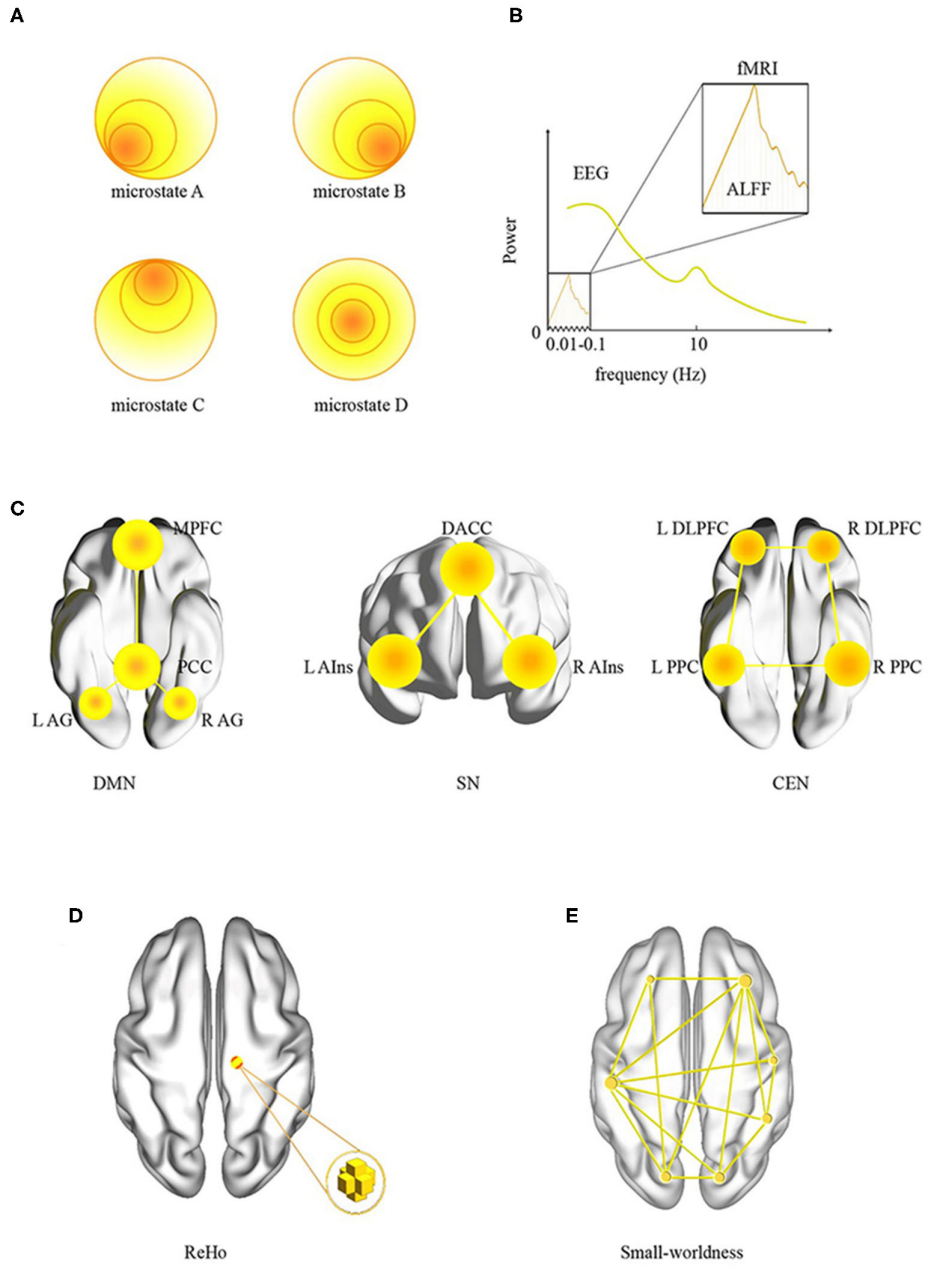
Data analysis methods in resting-state neuroimaging. **(A)** EEG microstates; **(B)** EEG and fMRI power spectra, notice the difference in frequency ranges for each modality; **(C)** large-scale brain networks: DMN, SN, and CEN, and their key regions; **(D)** ReHo; **(E)** small-worldness. ReHo, regional homogeneity; ALFF, amplitude of low-frequency fluctuation; L, left; R, right; DMN, default-mode network; MPFC, medial prefrontal cortex; PCC, posterior cingulate cortex; AG, angular gyrus; SN, salience network; DACC, dorsal anterior cingulate cortex; AIns, anterior insula; CEN, central executive network; DLPFC, dorsolateral prefrontal cortex; PPC, posterior parietal cortex.

As illustrated in Figures 1A,B, there are two common analysis methods for rsEEG: power spectrum analysis (PSA) and EEG microstate analysis. Power spectrum analysis is employed to calculate the EEG power of different frequency bands. Electroencephalography signals can be converted from time domain to frequency domain by Fourier transformation, and the rhythms associated with specific neural functions can be extracted and quantified. These rhythms are mainly δ (1–4 Hz), θ (4–8 Hz), α (8–13 Hz), β (13–30 Hz), and γ (>30 Hz) (12). Although some features of PSA are widely used in the researches of OSA, many other features, such as alpha peak frequency, left–right asymmetries, and scale-free properties, are rarely investigated in OSA-related studies.

Electroencephalography microstate analysis investigates brain activity at quasi-stable states of ~100-ms duration. Based on the clustering of the EEG topography over time, some recurring and stable topographies were identified during resting state (13, 14). Because the duration of the EEG microstate is similar to the duration of a single thought, it was assumed to represent the “atom” of thought (13). Currently, EEG microstate analysis is employed to explore brain changes in mental diseases such as insomnia (15), narcolepsy (16), and schizophrenia (17). However, for our knowledge, there is no study investigating the changes of EEG microstates in patients with OSA.

At present, PSA is the most common analysis method in rsEEG study of OSA. In contrast, many other advanced EEG analysis methods, such as source location (18), brain network analysis (19), and detrended fluctuation analysis (20), are rarely utilized for the studies of OSA. Meanwhile, these advanced analysis methods faced more difficulties in the diagnosis of OSA, because they have higher requirements for hardware, software, and experience. In the future, greater opportunity and challenge may lie in the application of these new methods for the study of OSA. Based on experience and existing research results, we believe that different research methods have different potentials in clinical application, PSA may have higher application value, and the clinical application value of microstate may be low.

### Resting-State fMRI

Magnetic resonance imaging was utilized to collect signals of the whole brain with various scanning sequences. Resting-state fMRI is mostly based on the sequence of echoplanar imaging, which reflects the low-frequency spontaneous oscillations (typically 0.01–0.08 Hz) of blood oxygenation level–dependent (BOLD) signals. Brain regions with synchronous BOLD oscillations constitute a large-scale brain network. For neuroimaging of OSA, some large-scale brain networks receive more attention, such as central executive network (CEN), default-mode network (DMN), and salience network (SN) (21). As illustrated in Figure 1C, we listed these three networks and their key regions in the brain.

Most of the previous rsfMRI studies of OSA focused on the local properties (Figures 1B,D), that is, the amplitude of low-frequency fluctuation (ALFF) and regional homogeneity (ReHo). The former measures the spontaneous fluctuations of BOLD signal, whereas the latter focuses on the similarity of the regional signals (22). An interesting topic is a relationship between the power spectrum of EEG and the power spectrum of fMRI. As illustrated in Figure 1B, the ALFF focused on a very slow oscillation in fMRI. In contrast, EEG has a wide range of spectrum. They may represent similar neural activity in the frequency range of 0.01 to 0.1 Hz.

Functional connectivity, another commonly used method in rsfMRI, focuses on the statistical correlation between signals in different brain regions. In fact, FC can be divided into two categories: first, seed-based analysis, a model-based region-of-interest (ROI) analysis, requires determining the ROIs in advance based on previous studies or other experiments. Then, a typical process is to calculate the correlation coefficient among the seed regions or with the whole brain (22). Second, independent components analysis (ICA), a model-free analysis, separates BOLD signals into multiple sets of spatiotemporal components. For spatial ICA, the component is spatially independent of each other and constructs a large-scale brain network (21). In recent years, dynamic FC, an extension of static FC analysis, has been developed in rsfMRI. The duration of signal for estimating dynamic FC is a little short, usually <40 s. Dynamic FC may be a new method for the study of OSA (23).

Graph theory analysis is occasionally adopted in OSA studies (24). It provides a relatively simple but powerful quantitative framework to describe whole human brain networks (25). For graph analysis, the nodes can be voxels, ROIs, or even large-scale brain networks. As illustrated in Figure 1E, small-worldness is a frequently discussed parameter. With a large clustering coefficient and small average shortest path length, whole brain is statistically imitated as a small-world network.

At present, a growing body of analysis methods has been developed and applied in the study of OSA, which will undoubtedly promote the further exploration of the mechanism of this disease. More importantly, the reliability and validity of these methods are still worth to verify. Besides, based on experience and existing research results, we believe that different fMRI research indicators have different clinical research potentials. For example, graph theory and FC may have lower clinical potentials, whereas ALFF and ReHo may have higher application value.

## Resting-State Neuroimaging of OSA

In order to systematically investigate the application of resting brain imaging in OSA, we conducted our search in the Google Scholar, Scopus, and PubMed databases in April 2020 to systematically explore studies using rsEEG and rsfMRI in patients suffering from OSA. The language screening standard of this article is English. The keywords were “(functional magnetic resonance imaging OR fMRI), (electroencephalography OR EEG), (resting state OR rest) and (sleep-related breathing disorders OR sleep apnea OR OSA).” A total of 50 studies were retrieved from the database. Subsequently, we excluded studies that included other technology (*n* = 3), reviews (*n* = 2), and studies that were not related to the main topic of the present review (*n* = 21). The final results included 1,616 participants, with the age range from 4 to 89 years. Among all studies, 8 were about rsEEG (Table 1), and 16 were about rsfMRI (Table 2). There are more studies based on rsfMRI when compared with rsEEG.

**Table 1:** Resting-state EEG studies of obstructive sleep apnea.

	**References**	**Number of P/C**	**AHI of P**	**Age of P/C**	**Spectrum change (patients vs. controls)**
1	Morisson et al. (26)	21/10	62.9 ± 26.1	44 ± 7/44 ± 6	↑δ, θ activity(frontal) ↑(δ + θ)/(α + β) (frontal, central, parietal, occipital, temporal)
2	Morisson et al. (27)	14/10	62.8 ± 25.8	45 ± 6.4/44.2 ± 6.1	↑δ absolute activity in OSA (frontal) ↑(δ + θ)/(α + β) (frontal, central)
3	Mathieu et al. (28)	(12/13) [young] (13 /14) [old]	46.9 ± 20.3 [young] /42.8 ± 24.7 [old]	38.2 ± 6.4/35.8 ± 8.9 62.2 ± 5.6/60.2 ± 6.4	↑(δ + θ)/(α + β) (frontal, central, parietal, occipital, temporal) in both young and old groups
4	Grenèche et al. (29)	12/8	66.1 ± 11.2	51.2 ± 2.5/49.4 ± 3.4	↑δ, θ, β power
5	Xiromeritis et al. (30)	(28/35/68)/30	10.4 ± 3(mild)/21.3 ± 4.1(moderate)/64.4(severe) ± 18.7(control)	(46.2 ± 11.4/51.3 ± 8.5/49.6 ± 10)/46.7 ± 11.8	↑θ, δ power (occipital, temporal, parietal)
6	Baril et al. (31)	12/12	51.2 ± 23.9	47.9 ± 13.7/44.4 ± 9.5	No change
7	D'Rozario et al. (32)	8/9	49.8 ± 24.7 46.62 ± 7.1	44.6 ± 8.4/27.8 ± 3.7	↑δ power
8	Zeidy Muñoz-Torres et al. (33)	22 [men]/21 [women]/0 [control]	49.8 ± 24.7 46.62 ± 7.1 [men]/53.87 ± 7.5 [women]	54.4 ± 2.4 [men]/57.3 ± 2.6 [women]	↓α power ↓β-γ power (frontal, central, moderate OSA, men vs. women) ↓δ power (frontal, central, occipital, severe OSA, men vs. women)

*P, patients; C, controls; AHI, apnea–hypopnea index (events/h); PSA, power spectrum analysis; ↑, increased; ↓, decreased; (δ + θ)/(α + β), slowing ratio*.

**Table 2:** Resting-state fMRI studies of obstructive sleep apnea.

	**References**	**Number of P/C**	**AHI of P**	**Age of P/C**	**Change (patients vs. controls)**
1	Zhang et al. (34)	24/21	54.7 ± 19.9	44.6 ± 7.4/40.6 ± 11.4	FC: ↑PCC(pDMN); ↓MPFC(aDMN), DLPFC(CEN)
2	Santarnecchi et al. (2)	19/19	36.3 ± 13	43.2 ± 8/41 ± 6	ReHo: ↑thalamus, somatosensory, motor ↓right temporal, parietal, frontal
3	Peng et al. (35)	25/25	60.6 ± 18.6	39.4 ± 1.7/39.5 ± 1.6	ReHo: ↑right posterior cerebellum, right cingulate gyrus, lateral lenticular nucleus, putamen, insula ↓nodes of DMN
4	Zhang et al. (36)	24/21	44.6 ± 7.4	44.6 ± 7.4/40.6 ± 11.4	↓FC between right AIns and the nodes of the DMN: MPFC, ACC, SFG, IPL, ITG
5	Li et al. (37)	25/25	60.0 ± 18.6	39.4 ± 1.7/ 39.5 ± 1.6	ALFF: ↓hubs of DMN
6	Taylor et al. (38)	19/17	6 ± 2(no or mild)/28 ± 2(moderate or severe)	58 ± 4/57 ± 4	No change
7	Park et al. (39)	67/75	35.6 ± 23.5	48.0 ± 9.2/47.1 ± 9.3	↓global efficiency, weighting clustering coefficient and nodal centralities
8	Park et al. (40)	69/82	35.6 ± 23.3	48.3 ± 9.2/47.6 ± 9.1	FC changes between insular and many brain regions, PFC, parietal, temporal, cingulate gyrus, basal ganglia, thalamus
9	Li et al. (41)	40/40	59.5 ± 20.9	38.6 ± 8.1/39.3 ± 7.5	↑FC between left IPL and right IPL, and between the MPFC and left and right IPL ↓right hippocampus and the PCC, MPFC, and left MTL
10	Li et al. (42)	36/40	56.5 ± 19.0	39.0 ± 8.1/38.8 ± 11.2	DC ↑lenticular nucleus, the putamen, posterior cerebellar ↓PCC, IPL, left SFG
11	Chen et al. (43)	30/25	62.5 ± 19.2	38.3 ± 8.4/39.5 ± 8.0	↑normalized characteristic path length, local efficiency ↓normalized clustering coefficient, small-worldness, global efficiency
12	Chen et al. (44)	45/45	58.7 ± 20.38	37.56 ± 8.86/37.84 ± 11.38	↑characteristic path length ↓normalized clustering coefficient, small-worldness, global efficiency ↓nodal centralities of DMN,SN,CEN
13	Chen et al. (45)	46/46	58.26 ± 20.37	20–60	↑nodal DC in the ventral medial PFC and the right parahippocampal cortex ↓clustering coefficient, local efficiency, and nodal centralities in the left PCC and DLPFC
14	Huang et al. (46)	29/26	33.67 ± 21.75	39.62 ± 9.95/ 34.46 ± 9.97	↑characteristic path length ↓clustering coefficient, local efficiency, global efficiency
15	Song et al. (47)	70/89	36.0 ± 23.3	48.3 ± 9.2/ 46.4 ± 9.2	↑FC among hippocampus, precuneus, PCC ↓FC nodes of DMN (IPL, AG)
16	Yu et al. (48)	40/40	60.1 ± 20.45	37.0 ± 8.74	↑FC among left DA, anterior lobe of the cerebellum, among left VA, left IFG and left STG, between right VA and left IFG ↓FC between right DA and right PFC

*P, patients; C, controls; AHI, apnea–hypopnea index (events/h); ICA, independent component analysis; ReHo, regional homogeneity; FC, functional connectivity; DC, degree centrality; ↑, increased; ↓, decreased; DMN, default-mode network; aDMN, anterior DMN; pDMN, posterior DMN; MPFC, medial prefrontal cortex; PCC, posterior cingulate cortex; CEN, central executive network; DLPFC, dorsolateral prefrontal cortex; AIns, anterior insula; ACC, anterior cingulate cortex; SFG, superior frontal gyrus; IPL, inferior parietal lobe; ITG, inferior temporal gyrus; MTL, medial temporal lobe; ODI, oxygen desaturation index; MOG, Middle occipital gyrus; SMA, Supplementary motor area; MCC, middle cingulate cortex; MTG, medial temporal gyrus*.

Previous studies mostly focused on the changes in brain activity in OSA patients during sleep. In this article, the resting state was defined as the rest state of wakefulness, and the changes in brain activity in the non-sleeping process were focused on.

### Application of rsEEG in OSA

Until now, only seven studies use rsEEG to explore the pathophysiological mechanism of OSA. The features that have been applied in OSA-related resting-state studies could be classified into two categories: the whole band spectrum and the slow-wave activity. According to the spectrum analysis, we found both absolute power and relative power were equally concerned. In addition, slow-wave activity has shown a consistently increasing trend. For the correlation between rsEEG features and PSG-derived OSA severity indicators, we found there are no positive findings.

**Figure 2:**
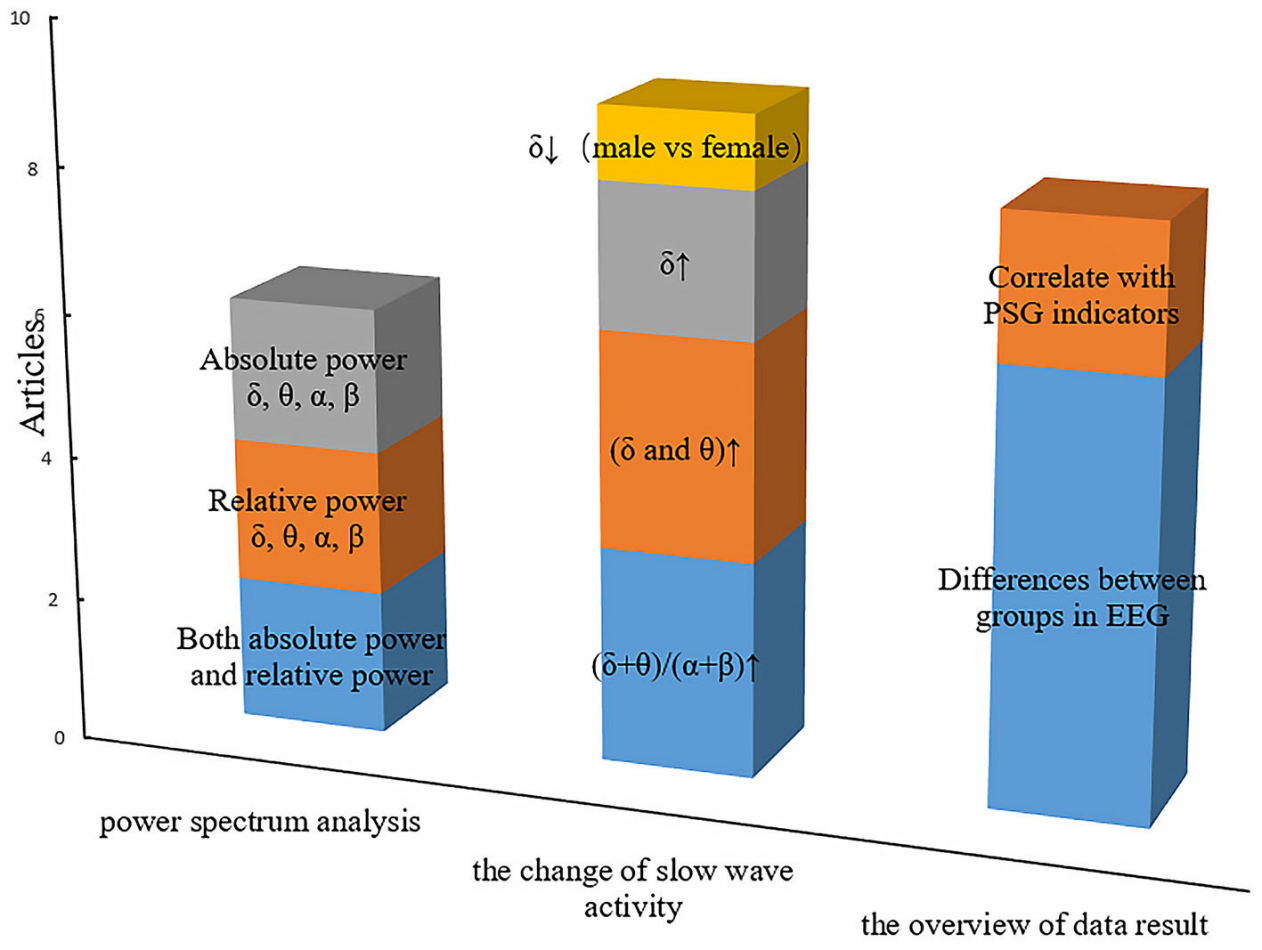
The primary features in resting-state EEG studies of obstructive sleep apnea. The height of the squares of different colors represents the number of articles related to special features.

Figure 2 summarizes the primary features that were frequently used in OSA studies. The height represents the number of articles for some common features. Both absolute power and relative power were equally concerned, and there were two articles that reported both of them. Another phenomenon is that slow-wave activity is widely reported, with eight articles in total. However, three articles have found an increase in the slowing ratio, three articles have found that both δ and θ are elevated, and only one article has found lower sigma power in men than women in the severe OSA group. In addition, two articles focus on the rise of δ, but not on the changes in θ. Rather than the correlation with PSG indicators, most studies are comparison between OSA patients and health control groups.

For rsEEG, spectrum powers in multiple bands are significantly altered for the OSA patients. Grenèche et al. found an increase in the δ, θ, and β powers, suggesting that maintaining wakefulness requires more cortical activities for OSA patients (29). Six rsEEG studies explored the difference of spectrum power between OSA patients and healthy control group (26, 27, 29–32). Among them, the enhancement of δ activity is a convergent phenomenon. However, very few studies, for example, Baril et al. (31), found no power differences between groups. Five studies found increased low-frequency activity (δ and θ) in the frontal and central regions in patients with OSA compared to healthy controls (26–28, 30, 32). Four studies calculated the slowing ratio (ratio of slow frequencies to fast frequencies) between OSA patients and normal controls. After regressing some confound factors such as weight, age, and education level, these studies found that OSA patients presented a steady increase in the slowing ratio (26–28, 30, 32). In addition, one study found gender differences in brain activity characteristics in OSA. δ, β, and γ powers are lower for men than for women (33). In summary, the increasing power of the low-frequency band, especially the δ rhythm in the frontal and the central areas, is a relatively stable pathological feature for OSA. We speculated that the slow-wave enhancement might be one of the essential criteria for OSA diagnosis and treatment in the future.

To further explore the physiological and psychological mechanisms under abnormal EEG activity in OSA patients, some researchers investigated the relationship between the features of rsEEG and the severity of OSA. Positive correlations between δ and θ relative power and apnea–hypopnea index (AHI) were revealed in patients with moderate to severe OSA (30). Another group reported that the slowing ratio in severe OSA patients was positively correlated with arousal index (ArI) and AHI (28). Not only the slowing ratio, some researchers even found that AHI and α power were positively correlated in OSA patients, and oxygen desaturation index (ODI) was positively correlated with θ and α power. In addition, they found that daytime alertness efforts were related to ArI, and daytime sleepiness was related to ODI (49). Other studies found no significant correlation between OSA severity and any rsEEG rhythm (26, 27, 49, 50). Only two studies found a significant correlation between subjective sleepiness and rsEEG power. And regrettably, the results were inconsistent. In one study, Epworth Sleepiness Scale (ESS) was positively correlated with the δ, θ, and α relative powers in eyes-open condition, and the similar correlation was identified with the δ and α relative powers in eyes-closed condition (32). Another study reported that the ESS was negatively correlated with δ and θ relative powers and positively correlated with α relative power, in eyes-closed and eyes-open conditions separately (30). In addition, no significant differences have been found between genders related to sleep and respiratory factors (33).

In summary, the current results of PSA of rsEEG were relatively consistent. A predominant phenomenon was the increased low-frequency activity, especially in the frontal and central regions. Furthermore, this increased activity was related to daytime sleepiness, which may be caused by long-term hypoxia at night. However, no robust association was found between various indicators of OSA severity and the power of different rsEEG bands. Because of the small number of literature (only eight), a large-scale comparison is impossible in our current review.

Current studies of rsEEG in OSA patients faced some common problems, such as a small number of samples and lack of application of PSG. More importantly, many advanced analyses require more electrodes, so the high-density EEG devices may be necessary. However, the collection of rsEEG data using high-density EEG is a heavy burden for patients, especially patients with severe OSA. It is worth mentioning that PSG cannot completely replace high-density EEG, because the latter has a large number of features and wide coverage of electrodes. For OSA patients, the future study may further consider EEG source imaging based on high-density EEG and may reveal more physiological information with high spatial resolution (18). Besides, the current studies on the resting-state of OSA patients focus on the differences between patients and normal people. Perhaps it may be considered to conduct research based on gender differences, age differences, and different high-risk inducers (such as drinking, obesity, etc.). At the same time, the changes in brain electrical activity of the same patient group in three different states of daytime resting-state, daytime sleepiness state, and night sleep state may also be very interesting.

### Application of rsfMRI in OSA

**Figure 3:**
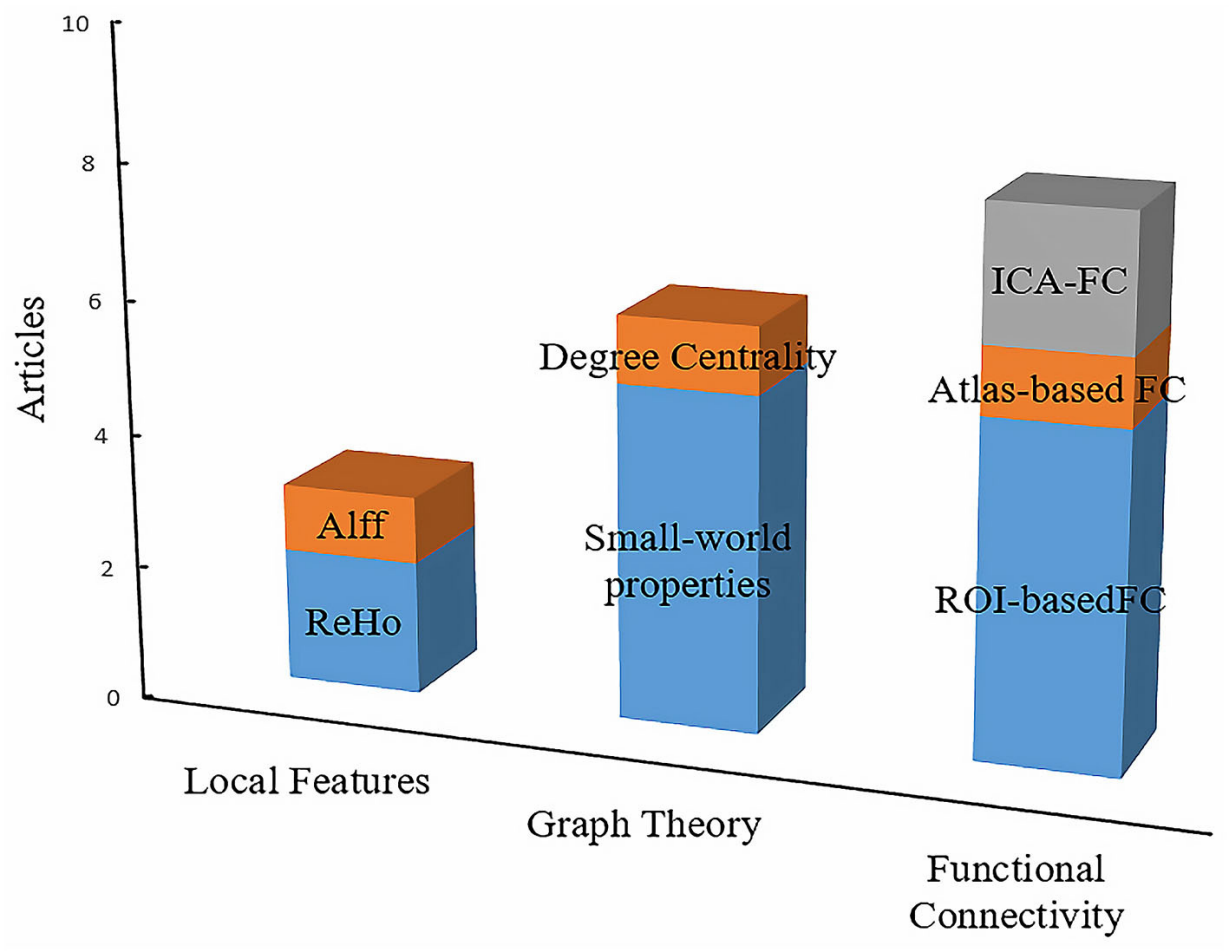
The primary features in resting-state fMRI studies of disorders of consciousness. The height of the squares of different colors represents the number of articles related to the contents of the square. In conclusion, the interpretation of the other column charts is consistent with the Figure 2.

The characteristics that have been applied in OSA-related resting-state studies could be generally classified into three categories: the local features, FC, and the graph theory. Figure 3 summarizes the primary features that were frequently used in OSA studies. There were three articles that used local features, such as ALFF and ReHo. In addition, we found that FC and ROI-based FC were widely employed to measure the aberrant brain synchronous activity of OSA patients. Graph theory is also increasingly utilized, and it is powerful for investigating the topological properties of the large-scale brain networks (45). In addition, there were three articles that used local features, such as ALFF and ReHo.

For FC analysis, large-scale brain networks, especially the DMN, SN, and CEN, were the focus of the rsfMRI study of OSA (51). The DMN consists of the posterior cingulate cortex (PCC), precuneus, medial prefrontal cortex (MPFC), inferior parietal lobe (IPL), hippocampus, and angular gyrus (AG) and can be further divided into two modules, namely, the anterior DMN and posterior DMN. The main characteristic of the DMN is that it is inhibited during the goal-oriented task, but highly active at rest (21, 22). The CEN mainly includes the dorsolateral prefrontal cortex (DLPFC) and posterior parietal cortex (PPC), and it is thought to be related to cognitive processes such as decision-making and working memory (36). The SN mainly includes the dorsal anterior cingulate and anterior insula (AIns), as well as some subcortical regions, such as the thalamus, striatum, and amygdala. Most of these brain regions are involved in emotions and goal-directed responses. During the execution of specific cognitive tasks, the above three brain networks always cooperate, and the moderation of the SN between the DMN and CEN may be the physiological mechanism of transition from resting state to cognitive processing state (52).

From the perspective of the large-scale brain networks, functional abnormalities of DMN network were often reported in rsfMRI studies of OSA. Six studies have reported abnormal internal connectivity of the DMN, including changes in global and local characteristics of the DMN, FC, and modulation structure (34, 35, 37, 41, 42, 45). Among them, the decrease in FC between the anterior DMN and other nodes of DMN is a relatively consistent result. In contrast, it is still controversial whether the FC within the posterior DMN is abnormal or not (34, 45).

Not only the DMN but also the CEN and the SN networks were reported that they had the abnormal FCs in OSA patients. A dominate change is the FC between the prefrontal lobe and insula. A study led by Zhang found reduced internal FC of DLPFC in OSA patients. In addition, Yu et al. found reduced FC between the right dorsal amygdala (DA) and right PFC (34, 48). Three studies have found that insula, one of the key brain regions of the SN, has impaired or even broken FC with many other brain regions, including prefrontal cortex (PFC), parietal lobe, temporal lobe, and cingulate gyrus (36, 39, 40). For example, a study suggested that the FCs between the right insula and multiple nodes of DMN were decreased. And the similar decrease can be observed in the FCs between the hippocampus and the dorsum medial thalamus, parahippocampal gyrus, and insula, which partly explained the declined working memory ability of OSA patients (36). In addition, the caudate nucleus has an abnormal FC with several nodes in the DMN, especially with the IPL and AG (47). In addition, abnormal FC was revealed in the amygdala subregion, as well as in the left DA and anterior cerebellum (including 4/5 vermis), left ventrolateral amygdala (VA), left inferior frontal gyrus (IFG), and left superior temporal gyrus (STG), and enhanced FC was identified between the right VA and left IFG (48). An analysis of ReHo indicators across the whole brain yielded similar results in the amygdala (2).

Graph theory was utilized occasionally in the rsfMRI studies of OSA. The main work came from the groups of Park, Chen, and others. In Park's study, he and his colleagues found the decrease in global efficiency, weighted clustering coefficient, and node attribute of whole-brain region in OSA patients (40). Compared to healthy controls, OSA patients showed decreased clustering coefficient and local efficiency, increased characteristic path length, and decreased node degree of left PCC and dorsal medial PFC, and the node degree of ventral PFC and right parahippocampal gyrus increased (44, 46). In addition, Chen et al. also found that changes in global and local network properties and changes in the properties of several major nodes reflect the abnormal connection between the three major networks and may be related to cognitive impairment (45). These studies are consistent with the results of previous studies on other indicators. Researchers speculated that chronic nocturnal hypoxemia leads to changes in small-world characteristics of patients' brain networks, which have certain effects on whole-brain function. Although the results of graph theory metrics are sometimes difficult to interpret from behavioral or pathophysiological points of view, the study of patients' small-world network properties and the evolution of their patterns may be one of the indicators to distinguish OSA severity.

In summary, the FC associated with the three large-scale brain networks and their major nodes is significantly altered for OSA patients. The decrease in the strength of connections between different networks may explain the decline in the related functions, whereas the enhancement of FC within some network nodes can be understood as functional compensation to some extent. The evidence from graph theory analysis, such as the increase in characteristic path length and the decrease in global efficiency, is also the evidence for the above opinion. Therefore, reduced FC between large-scale networks and enhanced FC within networks may be utilized as stable biomarkers of OSA disease, which is the distinctive evolution pattern of OSA's influence on brain function. In the future, with the support of more evidence, these FC-based biomarkers may be applied in the screening and diagnosis of diseases. In addition, the current research on OSA mainly focuses on the static local indicators and the calculation of the whole-brain network. Perhaps we can further focus on the dynamic changes of the above biomarkers and the more microscopic differences of these biomarkers in different frequency bands. At the same time, because it is difficult for OSA patients to stay awake for a long time, it is particularly important to explore the transition from wakefulness to daytime sleepiness in OSA patients.

### RsfMRI of OSA: Relationship With Severity of Disease

To better understand the relationship between abnormal neuroimaging features and the severity of the disease, investigating the relationship between direct physiology indicators of OSA and quality features of brain imaging is undoubtedly quite important. Physiological indicators from PSG are more representative of OSA severity, especially AHI and ODI. For example, a significant positive correlation between the connections of brain and ODI was found (48). Other researchers, such as Park, demonstrated that the function of the connection of the left insula and precuneus, IFG, cingulate central operculum, and IFG is positively correlated with AHI, and the connections in the left insula and bilateral sense–motor areas, left middle temporal gyrus, left middle temporal gyrus, left anterior central gyrus, right posterior hippocampus, and right cerebellum were negatively correlated with AHI (40). Zhang et al. found that the FC between the right AIns and MPFC was positively correlated with AHI, negatively correlated with the lowest saturation of blood oxygen (Sao_2_), and the internal FC of the right DLPFC was negatively correlated with AHI (36). The right DLPFC internal FC was negatively correlated with AHI (34). A Canadian group suggested that the FC of a small number of voxels in the right AIns is positively correlated with AHI (38), and decreased FCs between the right and left hippocampus and bilateral dorsum medial thalamus were negatively correlated with AHI (47).

Topological properties of brain networks were also correlated with AHI; for example, normalized clustering coefficient was negatively correlated with AHI, and normalized characteristic path length was positively correlated with AHI (43). In a word, according to the physiological indicators of PSG, we can verify the key role of PFC and insula in OSA research, which are closely related to the severity of OSA. Further research on PFC and insula may provide help for a more detailed diagnosis of OSA severity.

## Current Status and Future Directions

Currently, based on resting-state neuroimaging, the abnormal brain functions of OSA patients were detectable by both the EEG and fMRI techniques. However, clinical research is more challenging to control extraneous variables, and there are still many uncertainties in the study of the brain function of OSA patients. Therefore, many assumptions and research results need to be verified by more rigorous experiments and more exploratory discoveries. In the following part, the future directions of OSA research were discussed with several perspectives, including OSA-related interventions, the contribution of simultaneous EEG-fMRI, machine learning, and comorbidity.

### OSA-Related Interventions

Continuous positive airway pressure (CPAP) is the most effective and widely used treatment of OSA. The mechanism of CPAP is elevating collapsed upper airway tissue to prevent airway obstruction (53). It is known that CPAP recovers both AHI and oxygen saturation in OSA patients. However, the evidence from resting-state neuroimaging is very limited. A Korean group found that the slowing ratio in the whole-brain area decreased after treatment (53). Several studies have pointed out that the main finding on the rsEEG is the reduction of θ power (27, 30, 49). For δ power, a study led by Xiromeritis et al. (30) found a significant increase in δ power after treatment, whereas another study found the contrary conclusion (27). We speculate that different phases of CPAP intervention, potential individual variations, and patient tolerance may be the key reasons for the observed different response in resting-state neuroimaging. On the one hand, rsfMRI study that recorded both pre- and post-CPAP treatment is scarce. On the other hand, the current results of rsEEG are always inconsistent. Thus, it is worth discussing whether FCs within any large-scale brain network or any small-world network properties are improved after treatment. Furthermore, differences in treatment duration and frequency should be fully considered for the influence of CPAP treatment.

In conclusion, the various indicators of resting-state brain imaging can be used as a criterion for evaluating the efficacy before and after the intervention and promote a more in-depth understanding of the physiological mechanisms of the disease. Therefore, the application of resting-state neuroimaging is worthy of expectation for the development of clinical interventions.

### EEG-fMRI

Obstructive sleep apnea affects patients' daytime behavior by affecting their sleep quality. Therefore, it is undoubtedly necessary to research OSA patients' sleep states, such as sleep quality, sleep structure, and the brain activity during sleep, which requires simultaneous PSG recording during fMRI scanning. Combined with the high spatial resolution of fMRI and the high temporal precision of EEG, simultaneous EEG-fMRI provides evidence from two perspectives, electrophysiology and blood oxygen metabolism, to explore abnormal brain activity in patients with OSA (54).

With the development of experimental facilities, such as MRI, the combination of conventional MR hardware and advanced scan sequences makes the repetition time reduce to 400 ms or even less. Previous studies have discussed the temporal correlation between EEG waveform and BOLD signal (22), suggesting that there are similarities between the characteristics of brain network patterns and spontaneous oscillations of α rhythms. Simultaneous EEG-fMRI may help researchers better understand the physiological mechanisms of OSA disease. And if we can find a certain relationship between the data of the two modalities on OSA, it will be of great reference value for the screening and diagnosis of diseases and even the subsequent intervention. However, because of the small number of relevant experimental studies, clinical application of this technology in diseases other than epilepsy still needs more exploration and attempts.

### Machine Learning

Most of the existing studies identify or classify OSA patients by the characteristics of the EEG changes produced by the patients during the sleep stage. In addition, the signal of rsEEG indicates the abnormal electrophysiological activity of patients in different frequency bands (55). Therefore, the slowing ratio calculated by PSA may be used to screen OSA patients before routine medical diagnosis in the future (56). A related short period of rsEEG measurement (usually ~5 min) can greatly reduce the workload of routine PSG diagnosis and save medical resources. However, its calculation and the norm for health people need to first be constructed.

In the field of diagnosis of clinical diseases, it becomes increasingly popular to achieve high-efficiency auxiliary diagnosis by the technology of machine learning. If this technology becomes established as part of routine clinical diagnosis, the resting-state neuroimaging may become an immensely valuable tool for clinical practice. Resting-state fMRI can measure patients' brain FC and also can further measure the small-world network parameters and topological properties. Multivoxel pattern analysis (MVPA) using algorithms, such as linear discriminant analysis and support vector machine, can more accurately distinguish the differences in activation patterns of brain regions in different states (57). By combining the MVPA and the resting-state information, patients and normal people can be distinguished (58). Similar work has been done in other diseases to identify some effective biomarkers in patients with Alzheimer disease (AD) (59), bipolar disorder, and major depressive disorder (58). For example, a noteworthy study evaluated four major features by analyzing abnormal resting-state FC and achieved a classification accuracy of 76.1% in distinguishing major depression from healthy controls (60). Therefore, the development of the above technology may play a role in the rapid diagnosis of OSA and the classification of subtypes in the future.

### OSA-Related Comorbidity

For the middle-aged and elderly, impairment of daily function due to cognitive impairment may have a significant impact on the quality of life, whether it is caused by OSA or its associated complications. Among them, the decline in the attention, executive function and working memory ability of the elderly is particularly obvious (61). Evidence from fMRI shows that the decreased activation of the anterior cingulate gyrus, dorsal frontal cortex, and PPC in patients with OSA leads to decreased working memory performance (62). The decrease in cingulate gyrus activity may be related to the execution of attention. Therefore, for the study of OSA patients, identifying the specific effect of comorbidities is very important. Besides, the interactions among the comorbidities and OSA are other important question. At present, some literature has reported the association between OSA and AD (63) or related biomarkers (64). Notably, because OSA patients tend to have multiple complications, it is difficult to fully control each of these complications in clinical practice, making it difficult to distinguish the neural substrate of cognitive impairment. Despite the difficulties, some studies also have tried to explore this aspect. After specifically recruiting OSA patients without complications, a study found that there was no significant difference in cognitive performance between the patient group and the healthy control group (65). Although this study suggests that OSA patients' cognitive impairment may not originate from OSA itself, it is difficult to control the influence of intelligence, education, and other confound factors. And some of the comorbidities may be caused by damage to brain areas caused by chronic hypoxemia and poor sleep quality. Therefore, the traceability of cognitive impairment may also be closely related to the severity of OSA, and the complex interactions between them are difficult to distinguish and quantify. Thus, the above conclusions require a lot of research and large sample studies.

## Conclusions

This article summarizes the literature on rsEEG and rsfMRI studies of OSA so far and provides some suggestions for the better intervention of OSA deterioration and alleviating its impairment on cognitive functions. In summary, evidence from the rsEEG focused on the increased power of δ and θ in the frontal and central regions of the patient, whereas evidence from rsfMRI mainly found functional abnormalities within and between the three large-scale brain networks, that is, the DMN, the SN, and the CEN.

In terms of intervention therapy, we need to pay more attention to the following aspects: reducing the diagnosis cost of patients and improving the unhealthy lifestyles of patients. In terms of the physiological mechanism of diseases, the existing rsEEG research focuses more on the specific rhythms and the abnormity of power, but lacks the research on the connections in various brain regions, so methods such as functional connection and EEG source localization are required. Meanwhile, most of rsfMRI studies pay attention to abnormal activity of large-scale brain networks, and less research has related these abnormalities with the severity of the disease. In addition, the existing studies have not combined the evidence of both EEG and fMRI to explain the pathology of OSA. It is expected that this review will promote our understanding of the resting-state characteristics of neuroimaging studies in OSA.

## Author Contributions

XL, WZ, and XW contributed conception and design of the study. YW performed the statistical analysis and wrote the first draft of the manuscript. XC wrote sections of the manuscript. All authors contributed to manuscript revision, read, and approved the submitted version.

## Conflict of Interest

The authors declare that the research was conducted in the absence of any commercial or financial relationships that could be construed as a potential conflict of interest.
